# Emergency department presentations because of atrial fibrillation: too many, too soon

**DOI:** 10.1007/s12471-021-01563-w

**Published:** 2021-03-30

**Authors:** R. Pisters, L. Voorhout, M. E. W. Hemels

**Affiliations:** 1 Department of Cardiology, Rijnstate Hospital, Arnhem, The Netherlands; 2 Department of Cardiology, Radboud University Medical Centre, Nijmegen, The Netherlands

‘What is happening? What should I do? Whom should I consult?’ You can almost hear your patients’ thoughts as their everyday lives are abruptly turned upside down by new-onset atrial fibrillation (AF). The million dollar question for patients familiar with paroxysmal AF is more likely: ‘Should I stay or should I go?’ That is, stay put and wait for the storm to pass or seek shelter at the emergency department (ED). At present, patients flock to the ED because for too long we have tricked ourselves into believing that *pursuing *sinus rhythm is always the holy grail. It proves not to be the case: not regarding mortality [1], not for long-term stroke risk [2] and also not for symptom management in recent-onset AF [3].

In this issue of the *Netherlands Heart Journal*, Pluymaekers and co-workers [4] provide a clinical perspective to suppress our false intuition to actively restore sinus rhythm in all patients with recent-onset AF. They do so by focusing on differences in clinical characteristics between patients with and without early spontaneous conversion. This was defined as a return to sinus rhythm without active pharmacological or electrical cardioversion within the arbitrary ‘early’ window: either en route to or within 3 h following presentation at the ED. As such, early spontaneous conversion occurred in 1 in 6 patients (16.8%) with two-thirds of patients (10.6%) already in sinus rhythm within 1 h and a median time to spontaneous conversion of 32 min. Before discussing potentially relevant characteristics, let us take a moment for a pragmatic point of view. By the time the laboratory results become available, the vast majority of patients with ‘early’ spontaneous conversion will already have sinus rhythm. This virtually abolishes the need for clinical determinants to engage in shared decision making at this point in time.

Corroborating the results from prior studies, Pluymaekers et al. found symptoms with a duration of less than 24 h to be the strongest predictor of spontaneous conversion. They observed a median time between onset of complaints and discharge from the ED of 4 h and 11 h, respectively, in patients with and without early spontaneous conversion. Therefore, one could argue that most patients visited the ED way too soon. What, in general, is missing are the factors driving these patients to seek immediate medical attention. Herein we must not underestimate our own, in hindsight, wrong doing by instructing patients and healthcare providers alike to instantly contact the ED.

Of further interest is the observation that in more than 50% of all patients who did not have an early spontaneous conversion, a rate control strategy was chosen. Thus, rather than reaching the full potential of our most sophisticated, highly equipped and staffed department, one could say we abuse it for something which could easily be done in a less expensive setting. Surely, critics will argue the generalisability of such a wait-and-see approach. True, it does not apply in all cases. But that is beyond the point. It is applicable to a large group of patients. In the study by Pluymaekers et al. patients known to have AF recurrences lasting longer than 48 h were excluded from the analyses. Moreover, those with signs of an acute coronary syndrome or heart failure were excluded as well. Keep in mind that 28% of patients nevertheless complained of dyspnoea and 20% experienced chest pain. In patients with known AF it is easier to judge if such symptoms are arrhythmia correlated or indicative of underlying conditions requiring urgent medical attention. Thus, even if we were to adopt an approach which allows all new-onset AF patients to present to the ED this would dramatically reduce the number of presentations by 66%.

In the end, we have an obligation to guide our patients through the storm. This calls for a prominent and frequent discussion of the key questions with our patients: ‘Why does AF (re)occur?’, ‘What can be done to prevent this?’ and ‘What true harm can it cause?’ In other words: attention to and education of our patients, not tunnel vision focused on immediate restoration of sinus rhythm. Also, this must—and can—coincide with our societal responsibility to ensure the healthcare system is accessible and affordable to everyone. Keep mindful of the purpose and use of our (ED) resources. Be prudent in advising patients with uncomplicated episodes of recent-onset AF to seek immediate shelter at the ED. Instead, we should empower our patients, redefine our network capabilities, i.e. involve the patients’ general practitioners, and prepare for a recurrence of AF: what to expect, what best to do and whom to consult first.
